# The NLRP3 Inflammasome as a Pathogenic Player Showing Therapeutic Potential in Rheumatoid Arthritis and Its Comorbidities: A Narrative Review

**DOI:** 10.3390/ijms25010626

**Published:** 2024-01-03

**Authors:** Po-Ku Chen, Kuo-Tung Tang, Der-Yuan Chen

**Affiliations:** 1 Rheumatology and Immunology Center, China Medical University Hospital, No. 2, Yude Road, Taichung 40447, Taiwan; pago99999@gmail.com; 2 College of Medicine, China Medical University, Taichung 40447, Taiwan; 3 Translational Medicine Laboratory, Rheumatology and Immunology Center, Taichung 40447, Taiwan; 4 College of Medicine, National Chung Hsing University, Taichung 402202, Taiwan; dirac1982@vghtc.gov.tw; 5 Division of Allergy, Immunology, and Rheumatology, Taichung Veterans General Hospital, Taichung 40705, Taiwan; 6 Faculty of Medicine, National Yang-Ming University, Taipei 112304, Taiwan; 7 Institute of Medicine, Chung Shan Medical University, Taichung 40201, Taiwan

**Keywords:** NLRP3 inflammasome, comorbidities, pathogenic player, therapeutic potential, rheumatoid arthritis

## Abstract

Rheumatoid arthritis (RA) is an autoimmune inflammatory disease characterized by chronic synovitis and the progressive destruction of cartilage and bone. RA is commonly accompanied by extra-articular comorbidities. The pathogenesis of RA and its comorbidities is complex and not completely elucidated. The assembly of the NOD-, LRR- and pyrin domain-containing protein 3 (NLRP3) inflammasome activates caspase-1, which induces the maturation of interleukin (IL)-1β and IL-18 and leads to the cleavage of gasdermin D with promoting pyroptosis. Accumulative evidence indicates the pathogenic role of NLRP3 inflammasome signaling in RA and its comorbidities, including atherosclerotic cardiovascular disease, osteoporosis, and interstitial lung diseases. Although the available therapeutic agents are effective for RA treatment, their high cost and increased infection rate are causes for concern. Recent evidence revealed the components of the NLRP3 inflammasome as potential therapeutic targets in RA and its comorbidities. In this review, we searched the MEDLINE database using the PubMed interface and reviewed English-language literature on the NLRP3 inflammasome in RA and its comorbidities from 2000 to 2023. The current evidence reveals that the NLRP3 inflammasome contributes to the pathogenesis of RA and its comorbidities. Consequently, the components of the NLRP3 inflammasome signaling pathway represent promising therapeutic targets, and ongoing research might lead to the development of new, effective treatments for RA and its comorbidities.

## 1. Introduction

The pathology of rheumatoid arthritis (RA) is characterized by an infiltration of macrophages, B cells, and T cells, synovial hyperplasia, and the progressive destruction of cartilage and bone, with resultant joint deformities [1,2]. It affects approximately 1% of the population in developed countries [3]. The exact etiopathogenesis of RA is not fully understood, and the proposed causes include genetic factors, various infections, and immune dysregulation with the excessive production of proinflammatory mediators such as cytokines and chemokines [4,5,6,7,8]. It has recently been established that the dysfunction of innate and adaptive immunity is a critical etiological factor in the development and maintenance of RA [7,9,10]. The pathogenic alterations in innate and adaptive immunity are potential targets for therapeutic intervention in RA [7,9,10]. Besides the typical involvement of joints, RA is often associated with other systemic organ diseases and is complicated by comorbidities and organ dysfunction due to the chronic inflammatory process [11]. It has been estimated that up to 80% of RA patients have one or more comorbidities, resulting in a shortening of one’s lifespan [12,13]. RA-related comorbidities mainly include atherosclerotic cardiovascular disease (ASCVD), osteoporosis (OP), and interstitial lung disease (ILD) [14,15,16,17,18,19,20]. The presence of comorbidities may affect RA disease activity, become a barrier to optimal disease control, and lead to an impairment in quality of life (QoL) [21,22]. RA-related comorbidities are often sub-optimally managed, and the effective treatment of RA-related comorbidities is an unmet need for rheumatologists in clinical practice.

Nucleotide-binding domain leucine-rich repeat-containing receptors (NLRs) containing a pyrin domain (NLRPs), the major components of inflammasomes, play a pathogenic role in innate immunity and inflammation [23,24,25]. The NLRP3 inflammasome, a supramolecular cytoplasmic complex, may respond to stimuli such as adenosine triphosphate (ATP) and then recruit caspase-1, which cleaves pro-IL-1β and pro-IL-18 into their active biologic forms [26,27,28]. It is established that NLRP3 inflammasome dysregulation causes autoinflammatory diseases (AIDs) [29,30,31,32]; such dysfunction may similarly contribute to RA pathogenesis [10,33,34,35,36,37,38,39,40,41,42,43], although the underlying mechanisms are not fully elucidated.

Targeting the complex pathogenic factors in RA, various emerging new agents are available for the treatment of this disease [44,45]. Recent guidelines for RA treatment rank Janus kinase inhibitors (JAKi) or targeted synthetic biologic disease-modifying anti-rheumatic drugs (tsDMARDs) in parallel with biologic DMARDs (bDMARDs) as the options for patients who are refractory to initial conventional synthetic DMARDs (csDMARDs) therapy [46,47,48]. Nevertheless, a proportion of RA patients still fail to respond to current therapies [49,50,51]. With the high cost of b/tsDMARDs and their associated increased infection risk, alternative or add-on therapeutic agents targeting the immune or inflammatory responses are worth exploring.

Supporting the significance of the NLRP3 inflammasome in the pathogenesis of RA and its comorbidities, increasing clinical and pre-clinical evidence has revealed the components of the NLRP3 inflammasome as potential therapeutic targets in RA [43,52,53,54]. This review aims to summarize the current research evidence on the pathogenic role of the NLRP3 inflammasome signaling pathway and its clinical implications as the therapeutic target in RA and its comorbidities.

## 2. Materials and Methods

### 2.1. Literature Search

**Figure 1:**
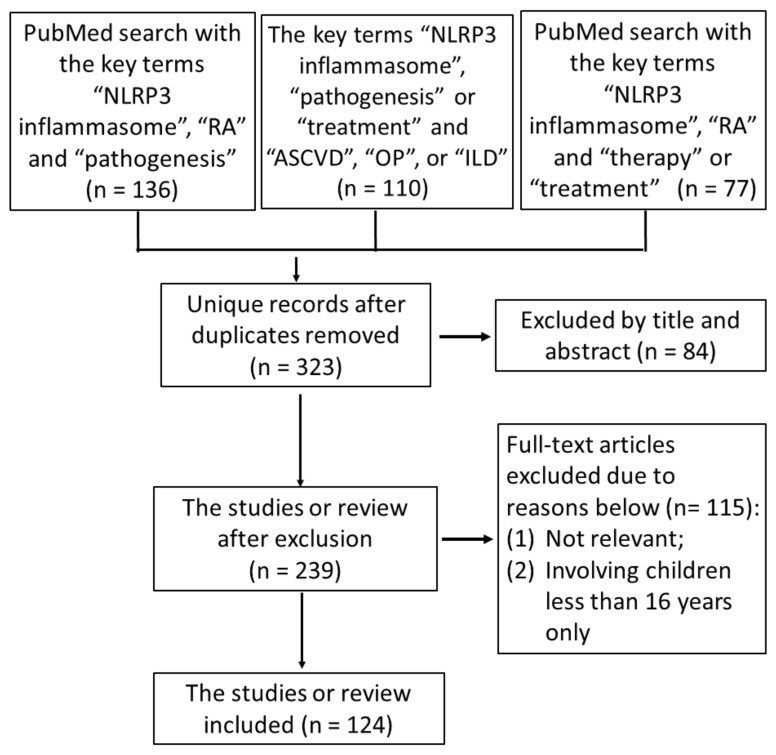
The flow diagram of the literature selection process (Search conducted on 31 October 2023). Duplicates and manuscripts with incomplete data have been excluded. RA: rheumatoid arthritis; NLRP3: nucleotide-binding domain leucine-rich repeat-containing receptors (NLRs) containing a pyrin domain-3; ASCVD: atherosclerotic cardiovascular disease; OP: osteoporosis; ILD: interstitial lung disease.

This review focuses on the updated research regarding the NLRP3 inflammasome as a pathogenic player and its therapeutic potential in RA. We searched the MEDLINE database using the PubMed interface and reviewed English-language literature as of 31 October 2023, from 2000 to 2023. The search keywords included pathogenesis, innate immunity, adaptive immunity, immune response, inflammation, NLRP3 inflammasome, proinflammatory cytokines, pyroptosis, IL-1β, IL-18, RA, RA-related comorbidities, ASCVD, OP, ILD, clinical implication, and therapeutic potential. Duplicates and manuscripts with incomplete data were excluded. The details of the search strategy are illustrated in Figure 1.

### 2.2. Study Selection

Three authors (PK Chen, KT Tang, and DY Chen) independently assessed the titles and abstracts from the search results and retrieved the relevant full-text articles. Two authors (KT Tang and DY Chen) independently evaluated the full-text articles for eligibility. Articles were selected if they (1) were probably relevant to the pathogenic role of the NLRP3 inflammasome in RA and its comorbidities, and (2) were potentially relevant to its therapeutic potential in this disease, including clinical trials, cohorts, case reports, and case-control studies.

### 2.3. Data Extraction

The authors extracted data from these studies electronically. Information regarding innate immunity, adaptive immunity, immune response, pathogenesis, the NLRP3 inflammasome, proinflammatory cytokines, pyroptosis, IL-1β, IL-18, clinical implications, therapeutic potential, RA, RA-related comorbidities, ASCVD, OP, or ILD was recorded from each study. The influence of relevant drugs, including small molecule inhibitors, natural products, corticosteroids, csDMARDs, bDMARDs, tsDMARDs, and targeted therapeutics for the NLRP3 inflammasome in RA and its comorbidities, was also documented.

## 3. Etiopathogenesis of RA and Its Comorbidities

The pathogenesis of RA is multifactorial and complex, including environmental factors, genetic variables, and immune dysregulation such as inflammasome activation and cytokine-mediated inflammation [1,2,3,4,5,6,7,8,55,56]. Kolly et al. demonstrated that the components of the NLRP3 inflammasome were highly expressed in the synovia of RA patients [33]. Recent evidence suggests that the percentages of CD4^+^ T cells with activated caspase-1 are significantly higher in RA patients compared with normal controls. The pharmacological and genetic inhibition of the DNA repair nuclease MRE11A may cause mitochondrial dysfunction in CD4^+^ T cells, leading to NLRP3 inflammasome assembly, caspase-1 activation, and pyroptosis in RA CD4^+^ T cells [57]. Increasing evidence indicates that the NLRP3 inflammasome plays a critical role in the pathogenesis of RA [10,46,47,48,49,50,51,52,53,54,55].

Atherosclerosis is a chronic inflammatory process that leads to vascular atheromatous plaque buildup and the development of full-blown ASCVD [58]. The high ASCVD burden in RA patients [59,60] may result from a combination of traditional risk factors, disease-specific factors, chronic inflammation, genetic components, and the use of medications [61,62,63,64]. Variants of the gene encoding apolipoprotein (apo)E have been shown to be related to ASCVD risk in RA patients [65]. Among the particles of low-density lipoprotein (LDL), a lipoprotein class, the density, size, electric charge, and composition are varied. Supporting the lipid paradox hypothesis in RA [66], studies have shown an inverse correlation between RA-related inflammation and circulating levels of LDL cholesterol (LDL-C) [67,68]. Electronegative LDL, a naturally occurring LDL, exerts potent atherogenic effects in cells and animals without undergoing ex vivo oxidation [69]. Elevated circulating levels of L5, the most electronegative subfraction of LDL-C, have been observed in RA patients and may be a predictor of ASCVD in this disease [68]. A high L5 percentage was significantly associated with elevated expression of the gene encoding integrin CD11c, which was linked to carotid arterial plaque formation [70].

Osteoporosis (OP) or bone fragility arises from a complex interaction of traditional risk factors and disease inflammation in RA. RA patients have an elevated risk of OP or osteoporotic fractures compared to healthy control participants [18,71]. Using Mendelian randomization analysis, Yu et al. revealed that genetically determined RA was linked to estimated bone mineral density (eBMD) and fracture risk [72]. Similarly, osteoporosis risk could be causally increased by the presence of anti-citrullinated peptide antibodies (ACPA) in Asians [72,73]. The presence of ACPA, a prolonged RA disease duration, significant exposure to corticosteroids, decreased physical activity, or a history of low trauma fractures are the risk factors of OP or fragility fractures in RA [73,74].

ILD is the leading cause of mortality and the most common pulmonary manifestation of RA [20,75]. It is estimated that approximately 30% of RA patients have subclinical ILD, as shown by high-resolution computerized tomography (HRCT) scans [76]. Although the exact pathogenesis of ILD in RA remains unclear [75,76,77,78], smoking, male gender, older age, high titers of ACPA, disease duration, and positivity of the Human leukocyte antigen (HLA)-DR4 were probable risk factors for RA-ILD [78,79,80,81,82]. A study of the Western population revealed the MUC5B promoter variant rs35705950 as a genetic risk factor for developing RA-ILD, particularly in those with the usual interstitial pneumonia (UIP) pattern [83]. Shirai et al. identified rs12702634 at RPA3-UMAD1 as a risk variant for RA-ILD in the Japanese population [84]. Citrullination, a post-translational modification characterized by the conversion of arginine to citrulline, and the emergence of ACPA probably contribute to RA-ILD by releasing neutrophil extracellular traps [85]. Recently, Zhang et al. revealed the pathogenic role of IL-17 in murine pulmonary fibrosis and RA-ILD [86]. Air pollutants, such as the elements of particulate matter (PM) < 2.5μm in size (PM2.5), may trigger the development of ILD in genetically susceptible patients [87].

## 4. NLRP3-Inflammasome

### 4.1. NLRP3-Inflammasome Signaling in Immune Responses and Inflammation

The innate immune system encompasses the germline-encoded pattern recognition receptors (PRRs), including Toll-like receptors (TLRs) and NLRs [35,88,89]. The NLRP3 inflammasome is a cytoplasmic protein complex with a key role in the innate immune response and inflammatory reaction. The assembly of the NLRP3 inflammasome recruits and activates caspase-1, which induces the maturation and release of proinflammatory cytokines, including IL-1β and IL-18 [26,27,28]. The activation of the NLRP3 inflammasome also leads to the cleavage of gasdermin D (GSDMD) at the GSDMD-N terminus [28,90] and promotes a lytic form of cell death, pyroptosis, with pores formation in the cell membrane and the release of IL-1β and IL-18 [91,92,93]. The NLRP3 inflammasome signaling may play a critical role in both innate and adaptive immunity and act as a checkpoint in innate immunity to lead to skewed adaptive immune responses in autoimmune diseases [10].

### 4.2. NLRP3-Inflammasome Activation and Regulation in RA Pathogenesis

The dysregulation of the NLRP3 inflammasome is linked to a variety of inflammatory diseases such as RA [10,33,34,35,36,37,38,39,40,41,42,94]. Zhang et al. demonstrated that NLRP3 was highly expressed in the synovial proliferation and subchondral vasculitis areas in the paws of collagen-induced arthritis (CIA) mice compared to control mice [34]. NLRP3 mRNA levels were significantly elevated in the synovia of RA patients compared to OA patients [38]. Guo et al. also documented that the NLRP3 inflammasome was highly activated in the synovia from RA patients and murine models [39]. Recent studies have found that pyroptosis is involved in the occurrence and progression of RA, and large amounts of IL-1β and IL-18 are present in RA patients. In RA, complement C1q and pentaxin 3 (PTX3) in monocytes synergistically promote NLRP3 inflammasome over-activation and pyroptosis [40]. Wu et al. revealed that acid-sensitive ion channel-1a mediates chondrocyte pyroptosis in arthritis by promoting NLRP3 inflammatory vesicle assembly, caspase-1 expression, and IL-1β and IL-18 release [95]. Ca^2+^ and cyclic AMP are two key molecular regulators of the NLRP3 inflammasome [96]. Werner et al. demonstrated that increased extracellular Ca^2+^ ([Ca^2+^]_ex_) could induce inflammation through promoting NLRP3 inflammasome assembly and IL-1β release [42]. They further proposed that increased [Ca^2+^]_ex_, calciprotein particles, and proinflammatory cytokines drive a vicious cycle of inflammation and bone destruction in RA [42]. The stimulation of anti-citrullinated peptide antibodies (ACPA) could activate pannexin channels with the release of ATP and promote the NLRP3 inflammasome activation and IL-1β production in RA [41]. These observations suggest that the activation of NLRP3 inflammasome signaling and the ensuing overproduction of inflammatory cytokines are key to the pathogenesis of RA [35,36,37,38,39,40,41,42,97].

### 4.3. The Genetic Predisposition of the NLRP3 Inflammasome in RA

Nucleotide polymorphisms occur within the regulatory region of cytokine genes, and some are associated with an altered rate of gene expression. The NLRP3 inflammasome gene polymorphisms contribute to susceptibility, disease activity, or disease severity in RA [98,99,100,101]. The genetic variants of the NLRP3 inflammasome can also affect the therapeutic response to TNF-α inhibitors in RA patients [102,103,104].

### 4.4. The Involvement of NLRP3 Inflammasome Activation in RA-Related Comorbidities

Elevated cholesterol and genetic predisposition may trigger the activation of the NLRP3 inflammasome signaling pathway and promote the development and progression of ASCVD [105,106,107]. Karasawa et al. revealed that the crystallization of released cholesterol in the atherosclerotic plaque may activate the NLRP3 inflammasome with the production of IL-1β and IL-18 [105]. In contrast, the lack of caspase-1 exhibits a protective effect against the evolution of atherosclerotic lesions, further resonating with the causative association of NLRP3 inflammasome with atherosclerosis [105]. Rhoads et al. also demonstrated that the oxidized LDL (oxLDL) immune complex induced inflammasome activation through a more robust mechanism than oxLDL alone did [108]. An association study has identified the Q705K polymorphism(rs35829419) in the NLRP3 gene as a protective factor against the risk of developing infarction in females [109]. The C10X variants (rs2043211) in the CARD8 gene were related to the lower expression of CARD8 in carotid plaques in a Swedish cohort [110]. In a Chinese cohort, a variant (rs2043211) in the CARD8 gene was associated with ischemic stroke [111]. Kastbom et al. showed that genetic variants of the NLRP3 inflammasome were associated with ischemic stroke in Swedish patients with RA [112]. These observations suggest a close link between the NLRP3 inflammasome signaling pathway and ASCVD in inflammatory diseases such as RA.

Increasing evidence also supports the association between the NLRP3 inflammasome signaling pathway and OP [37,113]. In an ovariectomized (postmenopausal) OP rats’ model, the inhibition of the NLRP3 could increase the osteoblasts number and bone density, suggesting a pathogenic role of NLRP3 in OP [114]. The activation of the NLRP3 inflammasome contributes to the maturation of downstream proinflammatory cytokines, IL-1β and IL-18, and pyroptosis [26,27,28]. The pyroptosis of osteoblasts may participate in OP pathogenesis [115]. Lei et al. revealed that IL-17 could induce the pyroptosis of murine primary osteoblasts in the NLRP3-mediated pathway, which further promoted the release of IL-1β and the receptor activator of nuclear factor-kappa B ligand (RANKL) and exacerbated the progression of OP [116]. Interestingly, IL-1 may also participate in the TNF-α-mediated inflammatory bone loss [117]. He et al. reported an association of IL-1β haplotype with OP susceptibility in the Chinese Han population [118]. IL-18 could upregulate the production of key osteoclastogenic mediators and increase bone loss in RA [119].

There are strong mechanistic similarities between usual interstitial pneumonitis (UIP), the most common pattern of RA-ILD, and idiopathic pulmonary fibrosis (IPF) in RA. However, Lasithiotaki et al. revealed distinct NLRP3 inflammasome activation profiles between RA-UIP and IPF [120], with significantly higher levels of IL-1β and IL-18 in bronchoalveolar lavage fluid (BALF) from RA-UIP patients compared with IPF patients. Intracellular IL-1β levels were also augmented in RA-UIP BALF cells upon NLRP3 inflammasome stimulation [120]. These findings suggest that the NLRP3 inflammasome is involved in the pathogenesis of RA-ILD. Similarly, the aberrant activation of the NLRP3 inflammasome is observed in scleroderma-associated ILD [121,122]. Ramos-Martinez and colleagues demonstrated the enhanced activity of the NLRP3 inflammasome in the lungs of patients with anti-synthetase syndrome [123]. Hence, NLRP3 inflammasome and associated cytokines may participate in the pathogenesis of autoimmune diseases-related ILD.

## 5. Therapeutic Potential by Targeting the NLRP3 Inflammasome

### 5.1. Small Molecule Inhibitors

With increasing evidence supporting the importance of the NLRP3 inflammasome in RA pathogenesis [10,33,34,35,36,37,38,39,40,41,42], there has been significant interest in developing therapeutic agents targeting the components of NLRP3 inflammasome signaling [52,53,54]. One approach is to develop small molecule inhibitors that can block the activation of the NLRP3 inflammasome. As illustrated in Table 1, several compounds have been identified as inhibitors of the components of the NLRP3 inflammasome signaling pathway, including MCC950 [39,124,125], VX-765 [126], and disulfiram [127]. Guo et al. demonstrated that treatment with MCC950, a selective NLRP3 inhibitor, led to reduced joint inflammation and bone destruction in the murine RA model [39]. Another approach is to target the downstream products of NLRP3 inflammasome activation, such as the sophisticated pyroptosis pathway [127,128] and the production of pro-inflammatory cytokines. MCC950 could reduce macrophage infiltration and atherosclerotic lesion size through attenuating inflammation and pyroptosis in atherosclerosis murine models [129,130]. Li et al. reported that VX-765 could inhibit atherosclerosis in ApoE-deficient mice by modulating the pyroptosis of vascular smooth muscle cells [131].

### 5.2. Natural Products

Besides the small molecule inhibitors, several natural products exhibit anti-inflammatory effects through targeting the NLRP3 inflammasome signaling pathway (Table 1). Celastrol, a natural product isolated from *Tripterygium wilfordii*, has displayed therapeutic potential in inflammatory diseases, such as RA. One recent study showed the attenuating effects of celastrol on parvovirus B19-NS1-induced NLRP3 inflammasome activation in macrophages [132]. Jing et al. also revealed that celastrol inhibited inflammation by inhibiting the reactive oxygen species NF-κB-NLRP3 inflammasome axis and relieved RA symptoms [133]. Baihu-Guizhi decoction (BHGZD), a traditional Chinese-medicine-originated disease-modifying anti-rheumatic prescription, may reduce the disease activity of RA [134]. Li et al. reported that BHGZD could suppress the NLRP3 inflammasome activation and GSDMD-mediated pyroptosis by inhibiting NF-κB via TLR4/PI3K/AKT signaling in an adjuvant-induced arthritis-modified rat model [135]. Sulforaphene, extracted from radish seeds, has been demonstrated to suppress the M1 polarization of macrophages and reduce synovitis in the CIA murine model [136]. Osthole, a characteristic coumarin compound in the *Angelicae pubescentis* radix, can improve arthritis in the CIA rat model by inhibiting inflammation and oxidative stress [137]. Jiang et al. further showed that Osthole could inhibit NLRP3 inflammasome activation by regulating mitochondrial homeostasis [138]. Scropolioside B, isolated from a Tibetan medicine, *Scrophularia dentada* Royle ex Benth., could inhibit NF-κB activity, reduce NLRP3 expression, and suppress the maturation and release of IL-1β, suggesting its therapeutic potential in RA and its associated atherosclerosis [139]. Cao and colleagues demonstrated that Wedelolactone, derived from Eclipta alba, could ameliorate synovial inflammation, cardiac complications, and fibrosis by inhibiting the activation of the NF-κB/NLRP3 inflammasome pathway [140].

### 5.3. Disease-Modifying Anti-Rheumatic Drugs (DMARDs)

Hydroxychloroquine (HCQ), a known ion channel inhibitor, is a commonly used and effective treatment for RA. Schroeder et al. demonstrated that HCQ could inhibit Ca^2+^-activated K^+^ channels and suppress inflammasome activation [141]. Cui et al. also revealed that chloroquine inhibited the activation of the NLRP3 inflammasome in a murine model of hyperuricemic nephropathy [142]. Since IL-1β, an NLRPP3 inflammasome downstream cytokine, promotes synovial inflammation in RA patients, the IL-1β receptor antagonist anakinra has been approved for active RA patients unresponsive to csDMARDs [143]. Targeting IL-1β might also have therapeutic potential in RA-associated comorbidities. The Canakinumab Anti-inflammatory Thrombosis Outcome Study (CANTOS) study showed the benefit of canakinumab, an IL-1β blockade, in patients with previous myocardial infarction [144]. Given the pathogenic role of IL-18 in RA-associated OP [119], IL-18BP, an antagonist of IL-18, is speculated to be effective in the management of OP [145]. Mansoori et al. similarly revealed that high serum IL-18BP was associated with a low risk of osteoporosis in postmenopausal women [145].

### 5.4. Janus Kinase Inhibitors (JAKi)

Janus kinase inhibitors (JAKi) exert their therapeutic effects by blocking JAK/STAT-mediated signaling implicated in RA pathogenesis. Although tofacitinib, a JAK1/JAK3 inhibitor, is effective in RA treatment [49,50,51], the underlying mechanisms of drug action remain obscure. Yang et al. demonstrated that tofacitinib could restore the balance of γδTreg/γδT17 cells in RA by inhibiting the NLRP3 inflammasome [146].

### 5.5. microRNAs (miRNAs) and Stem Cells

Multiple miRNAs are shown to be involved in the post-transcriptional regulated expression of the NLRP3 inflammasome [147,148]. The miRNAs are short non-coding RNAs composed of approximately 20 to 24 nucleotides that mediate messenger (m)RNA cleavage, translational repression, or mRNA destabilization [149,150]. Xie et al. revealed an elevated expression of miRNA-33 as a positive regulator of the NLRP3 inflammasome in RA patients [151]. Yang et al. also showed that miRNA-30a might suppress the expression of the NLRP3 inflammasome in macrophages and regulate inflammation in RA [152]. A recent study revealed that miR-223 carried by bone marrow stem cells-derived exosomes can target NLRP3 and inhibit the activation of inflammasomes in macrophages and rat RA models [153]. Liao et al. also demonstrated that neutrophil-derived exosomal miR-223 could suppress NLRP3-inflammasome signaling and IL-18 production in macrophages in an in vitro assay [154]. Long non-coding RNAs (lncRNAs), the non-protein-coding transcripts greater than 200 nucleotides, have emerged as novel players in gene regulation [155,156]. The LncRNAs have been shown to be the key regulators of inflammatory responses [157]. Wang et al. reported that LncRNA *MIAT* (myocardial infarction-associated transcript) could downregulate IL-1β and TNF-α to suppress macrophage inflammation in the 774A.1 cell-based assay [158]. Recently, cell-based therapies using mesenchymal stem cells (MSCs) have been spotlighted as a promising strategy for the management of RA [159]. Shin et al. demonstrated that human umbilical cord blood-MSCs (hUCB-MSCs) could downregulate the activation of the NLRP3 inflammasome via a paracrine loop of IL-1β signaling in the CIA murine model [160], suggesting the therapeutic potential of hUCB-MSCs in RA treatment.

**Table 1:** The potential inhibitors of the components of the NLRP3 inflammasome in rheumatoid arthritis (RA) and its comorbidities.

Agents	Targets	Experimental Model and Mechanism	Diseases	References
	Small molecule inhibitors			
MCC950	The NACHT domain of NLRP3	Block ASC oligomerization and inhibit inflammation. (1) Reduce synovitis and cartilage erosion by inhibiting NLRP3 and caspae-1 activation in a CIA model. (2) Elevated liver enzymes in a phase II clinical trial.	RA, ASCVD, OP, ILD	[124,125,129,130]
VX-765	Caspase-1	Ameliorate the severity and progression of synovitis in a CIA murine model.	RA, ASCVD	[126,131]
Disulfiram	GSDMD	Inhibits pyroptosis and inflammatory cytokine release in both canonical and noncanonical inflammasome pathways.	RA and associated OP	[127]
	Natural products			
Celastrol (isolated from *Tripterygium wilfordii*)	Inhibit the ROS-NF-κB-NLRP3 inflammasome axis.	(1) Reduce synovitis through blocking the NF-κB pathway and inhibiting the NLRP3 inflammasome in a CFA-induced rat model. (2) Attenuate human B19 NS1-induced NLRP3 inflammasome activation in macrophages in U937 and THP-1 cells.	RA	[132,133]
Baihu-Guizhi decoction (BHGZD)	Inhibit TLR4/NF-κB/NLRP3 activation-induced pyroptosis.	Reduce synovitis as well as bone erosion and alleviate disease activity through inhibiting NF-κB via TLR4/PI3K/AKT signaling to suppress the NLRP3 inflammasome activation and GSDMD-mediated pyroptosis in an AIA-modified rat model	RA	[135]
Sulforaphene (extracted from radish seeds)	NLRP3	Suppress the M1 polarization of macrophages and reduce synovitis in a CIA murine model	RA	[136]
Osthole (extracted from *Angelicae pubescentis* radix)	AMPK agonist	Inhibit NLRP3 inflammasome activation by regulating mitochondrial homeostasis in a CIA rat model.	RA	[138]
Scropolioside B (isolated from *Scrophularia dentada* Royle ex Benth.)	NF-κB and the NLRP3 inflammasome pathway	Inhibit NF-κB activity, reduce NLRP3 expression, and suppress the maturation as well as the release of IL-1β.	RA and associated atherosclerosis	[139]
Wedelolactone, derived from Eclipta alba	NF-κB and the NLRP3 inflammasome	Ameliorate synovitis and cardiac complications via inhibiting the activation of the NF-κB/NLRP3 inflammasome pathway	RA and cardiac complication	[140]
	Disease-modifying anti-rheumatic drugs (DMARDs)			
Hydroxychloroquine or chloroquine	The second signal of NLRP3 activation	(1) Inhibit Ca^2+^-activated K^+^ channels, which leads to impaired inflammasome activation in THP-1 macrophages. (2) Inhibit NLRP3 inflammasome activation in a C57BL/6 mice model.	RA and associated ASCVD	[141,142]
Anakinra, a biological DMARD	IL-1β receptor antagonist	Inhibit the NLRP3 inflammasome downstream cytokine, IL-1β, in RA patients.	RA	[143]
Canakinumab, a biological DMARD	Monoclonal antibody targeting IL-1β	Reduce the rates of recurrent ASCVD, including myocardial infarction and stroke	RA and associated ASCVD	[144]
IL-18BP	IL-18 binding protein	Reduces Th17 cells, with the resultant inhibition of osteoclastogenesis, and induces osteoblasts formation.	RA and associated OP	[145]
Tofacitinib, a Janus kinase 1/3 inhibitor	NLRP3 inflammasome	Restore the balance of γδTreg/γδT17 cells by inhibiting NLRP3 inflammasome in a CIA model	RA	[146]
	Epigenetic regulators			
MiRNA-33 inhibitor	NLRP3 and caspase-1	MiR-33 impairs the mitochondrial oxygen consumption rate with increasing ROS and then upregulates NLRP3 inflammasome expression in macrophages in a CIA mice model	RA	[151]
MiRNA-30a	NLRP3	MiRNA-30a inhibits the NLRP3 inflammasome activation and reduces synovitis and bone damage in a TNFα-transgenic C57BL/6 mice model.	RA	[152]
MiRNA-223	NLRP3	MiRNA-223 from BMSCs-derived exosomes inhibits NLRP3 activation and the release of IL-β, TNF-α, and IL-18 in RAW264.7 cells by a luciferase reporter assay and rescue experiment	RA and associated ASCVD	[153]
LncRNA MIAT	IL-1β	LncRNA MIAT inhibited the expression of IL-1β and TNF-α and suppressed macrophage inflammation in a J774A.1 cell-based assay.	RA	[158]
	Allogenic mesenchymal stem cells			
hUCB-MSCs	NLRP3 inflammasome	Downregulate the activation of the NLRP3 inflammasome via a paracrine loop of IL-1β signaling in a CIA murine model.	RA	[160]

NLRP3: nucleotide-binding domain leucine-rich repeat-containing receptors (NLRs) containing a pyrin domain-3; NACHT: nucleotide-binding domain; TLR4: Toll-like receptor 4; PI3K: phosphatidylinositol 3-kinase; AIA: adjuvant-induced arthritis; CIA: collagen-induced arthritis; CFA: complete Freund’s adjuvant; hUCB-MSCs: human umbilical cord blood mesenchymal stem cells; BMSCs: bone marrow-derived stem cells; ATP: adenosine triphosphate; ROS: reactive oxygen species; GSDMD: gasdermin-D; IL-1: interleukin-1; Treg: regulatory T cells; TNF-α: tumor necrosis factor-α; MiRNA: microRNA.

Briefly, targeting the NLRP3 inflammasome signaling pathway represents a promising therapeutic strategy for RA and its comorbidities, and ongoing research would facilitate the development of novel and effective treatments for this disease.

### 5.6. The Side-Effects of Therapeutic Agents Targeting the NLRP3 Inflammasome Signaling Pathway

Based on the available reports, MCC950, an inhibitor of the NACHT domain of NLRP3, may exhibit adverse renal effects with the augmentation of renal inflammation and the emergence of glomerulosclerosis in diabetic individuals [161]. Disulfiram, a therapeutic agent targeting GSDMD, has rare side effects, including papular acne, pruritus, exfoliative dermatitis, myopathy, and cardiomyopathy [162,163,164]. Celastrol, an inhibitor of the ROS-NF-κB-NLRP3 inflammasome axis, may induce hepatotoxicity, ototoxicity, or cardiotoxicity [165]. The rare side effects of sulforaphane include dyspepsia and gastric upset [166]. HCQ, one of the csDMARDs, may have maculopapular rash, retinopathy, ototoxicity, myopathy, or cardiotoxicity [167,168,169,170]. The use of IL-β inhibitors, such as anakinra and canakinumab, may exhibit injection site reactions (ISRs) and increase the risk of infection [140,141]. The most common side effect of IL-18BP is ISRs [171]. The use of tofacitinib would increase the risk of infections, such as pneumonia, and herpes zoster [172]. Currently, no data regarding the side effects of therapies with non-coding RNAs are available.

## 6. Conclusions

**Figure 2:**
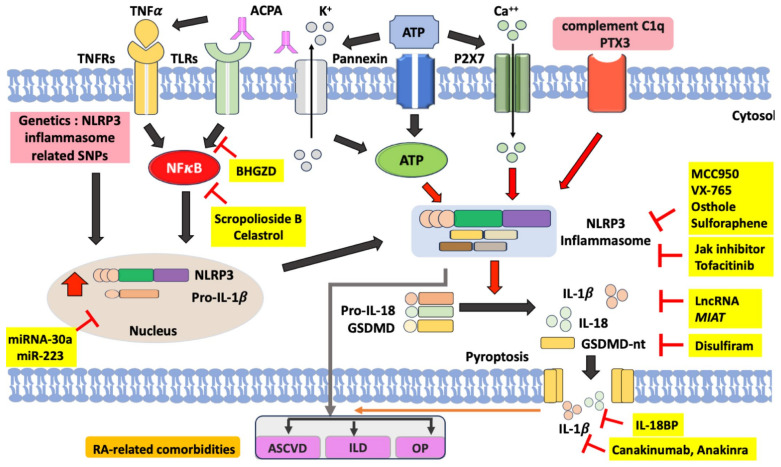
The proposed model for the pathogenic role of the NLRP3 inflammasome signaling pathway in RA and its clinical implications as the therapeutic potential. Several ligands that bind to TNFRs and TLRs can activate nuclear factor (NF)-κB. As a transcription factor, activated NF-κB can translocate into the nucleus and thereby activate the expression of NLRP3 and pro-IL-1β. As an endogenous ligand, anti-citrullinated peptide antibodies (ACPA) can promote NF-κB phosphorylation through binding to TNFRs and TLRs. In the second signal, extracellular ATP can bind to P2X7 and thereby lead to K^+^ efflux and extracellular Ca^++^ influx, which activate the NLRP3 inflammasome with the overproduction of the mature form of IL-1β and IL-18. The NLRP3 inflammasome activation also leads to the cleavage of gasdermin D, which promotes pyroptosis with the formation of pores in the cell membrane and the release of IL-1β and IL-18. ACPA can also activate the pannexin channel, resulting in ATP secretion and NLRP3 inflammation activation. In RA monocytes, complement C1q and pentaxin 3 (PTX3) synergistically activate the NLRP3 inflammasome and pyroptosis. Several compounds have been identified as inhibitors of the components of the NLRP3 inflammasome signaling pathway. MCC950, VX-765, osthole, and sulforaphane can inhibit the activation of the NLRP3 inflammasome. Tofacitinib, one JAKi, may restore the balance of γδTreg/γδT17 cells in RA by inhibiting the NLRP3 inflammasome. Disulfiram inhibits GSDMD and thereby blocks pyroptosis and the release of IL-1β and IL-18. Anakinra, an IL-1β receptor antagonist, blocks the effects of NLRP3 inflammasome downstream cytokine. Among natural products, both Baihu-Guizhi decoction (BHGZD) and celastrol can inhibit NLRP3 activation by blocking the NF-κB pathway. HCQ could inhibit Ca^2+^-activated K^+^ channels and then impair inflammasome activation. The microRNA-30a and miR-223 inhibit the expression of NLRP3 and inflammasome activation. Red arrows indicate activation of NLRP3 inflammasome. Block arrows trigger protein expression pathway. RA: rheumatoid arthritis; TNFα: tumor necrosis factor-α; TNFRs: TNFα receptors; TLRs: Toll-like receptors; ATP: adenosine triphosphate; PTX3: pentaxin 3; GSDMD: gasdermin D; IL: interleukin; ASCVD: atherosclerotic cardiovascular disease; OP: osteoporosis; ILD: interstitial lung disease.

With progressive insight into the pathogenesis of RA and its comorbidities, the role of the NLRP3 inflammasome is gaining importance in this disease [10,21,22,23,24,25,26,27,28,29,30]. Hence, the components of the NLRP3 inflammasome signaling pathway would be a promising therapeutic target in RA and its comorbidities [39,40,41]. Based on the available evidence, we summarized the data regarding the pivotal role of the NLRP3 inflammasome in the pathogenesis of RA, its comorbidities, and its therapeutic potential in Figure 2. Hopefully, this will lead to effective novel therapies for RA and its comorbidities.

## Data Availability

No data were used to support this review article.
